# Interviewer Administration Corresponds to Self-Administration of the Vision Impairment in Low Luminance (VILL) Questionnaire

**DOI:** 10.1167/tvst.11.4.21

**Published:** 2022-04-21

**Authors:** Jan Henrik Terheyden, Liza Mekschrat, Reglind A. D. Ost, Gamze Bildik, Moritz Berger, Maximilian W. M. Wintergerst, Frank G. Holz, Robert P. Finger

**Affiliations:** 1 Department of Ophthalmology, University Hospital Bonn, Bonn, Germany; 2 Institute for Medical Biometry, Informatics and Epidemiology, University Hospital Bonn, Bonn, Germany

**Keywords:** vision-related quality of life, administration, patient-reported outcome, PROM, psychometric assessment

## Abstract

**Purpose:**

To quantify the impact of the mode of administration (MOA) on scores of the Vision Impairment in Low Luminance (VILL) questionnaire.

**Methods:**

The VILL questionnaire was implemented using different MOAs (paper, interview, electronic), in addition to a demographical survey of adult participants recruited at an outpatient eye clinic, with the initial MOA being either paper or interview. Polytomous Rasch models were used to generate person measure scores for the three subscales of the VILL questionnaire (reading, VILL_R; mobility, VILL_M; and emotional, VILL_E). Measures of agreement among the different MOAs were calculated (self-administered paper/interview, self-administered paper/self-administered electronic, and interview/self-administered electronic). An age-matched analysis was performed to control for the impact of the initial MOA, administration interval, visual acuity, and self-reported hearing difficulties.

**Results:**

We included 309 participants (mean age, 63 ± 14 years; 61% female). Intra-class correlation coefficients were 0.930, 0.919, and 0.799 for paper versus interview assessment; 0.951, 0.959, and 0.916 for paper versus electronic; and 0.967, 0.955, and 0.907 for interview versus electronic assessment (VILL_R, VILL_M, and VILL_E, respectively). Mean differences were 0.35, 0.41, and 1.74 logits; 0.32, 0.18, and 0.68 logits; and 0.08, 0.22, and 0.63 logits, respectively. None of the mentioned factors significantly affected the results (corrected *P* ≥ 0.11).

**Conclusions:**

Paper, interview, and electronic MOAs of the VILL can be considered equivalent. Reporting across the main MOAs of self-administration (paper) and interviewer-administration was unaffected by better eye visual acuity and self-reported hearing difficulties.

**Translational Relevance:**

The results support use of the VILL questionnaire with flexible modes of administration.

## Introduction

In ophthalmology, there is growing awareness and popularity of outcome measures that are more patient centered than traditional assessments such as best-corrected visual acuity.^1^^–^^3^ Patient-reported outcome (PRO) measures of concepts such as vision-related quality of life (VRQoL) are attracting increasing interest in clinical routine, research, regulatory, and reimbursement contexts.^1^^,^^4^^,^^5^ PRO instruments are traditionally administered using paper-and-pencil questionnaires or in interviews, but the increasing digitization of medicine has made electronic modes of administration more relevant. However, PRO assessments that have been acquired via different modes of administration may only be comparable to a limited extent due to, for example, social bias introduced in an interview situation or technical restrictions of revisiting previous items in an electronic PRO.^6^^,^^7^ In ophthalmology, these sources of bias are further compounded by varying levels of visual acuity and ability to read and fill in any questionnaire due to varying levels of visual ability, the very construct many of the PROs are aiming to capture. This becomes even more relevant when considering the presentation of self-administered items, which may be impacted by color, contrast, and screen glare. Nevertheless, the impact of mode of administration has sparsely been investigated for ophthalmic PROs.^8^

The Vision Impairment in Low Luminance (VILL) questionnaire is a disease-specific ophthalmic PRO instrument that has recently been introduced. It focuses on patient impairment in low luminance and low contrast situations and has specifically been developed to capture the visual function deficit in age-related macular degeneration (AMD), one of the major blinding diseases in industrialized countries.^9^^,^^10^ The VILL has been administered in interviews or as a paper-and-pencil questionnaire, but these modes of administration have not been systematically compared. We have thus investigated the comparability of different modes of administration, including self-administration using paper and pencil or an electronic VILL version, as well as interviewer administration, hypothesizing that VILL scores obtained through different modes of administration are equivalent.

## Methods

### Participants

Adult participants with and without visual impairment were recruited from the Department of Ophthalmology, University of Bonn, Bonn, Germany, between 2018 and 2021. Ethics approval by the Institutional Review Board was obtained beforehand (approval ID: 130/16). The study adhered to the tenets of the Declaration of Helsinki, and all participants gave written informed consent prior to study inclusion. Exclusion criteria were cognitive impairment compromising the ability to consent or reply to the questionnaire, illiteracy, insufficient German language comprehension, any acute-onset diseases impairing vision 3 months before or at the time of participation, and withdrawal of consent.

### Questionnaire Administration

All participants were asked to provide demographic information and relevant medical history using a structured questionnaire. Three different modes of administration of the VILL questionnaire (VILL-33) were investigated:

• Self-administration via a paper-and-pencil form (“paper”)
• Interviewer administration (“interview”), administered via phone or in person
• Self-administration via an electronic form (“electronic”), using a tablet or desktop computer

Participants were required to complete at least two of these administration modes. Participants for whom only one mode of administration was available or with response intervals > 10 weeks between different modes of administration were excluded from analyses. Similarly, questionnaires with ≥50% missing responses were excluded from analysis. In addition to the VILL questionnaire, the participants were asked to self-complete a paper version of the German Low Luminance Questionnaire (LLQ-23). The LLQ is another instrument that assesses mainly night vision impairment.^11^^,^^12^ All interviews conducted at the clinic took place in a quiet, adequately illuminated environment by trained interviewers. Participants were offered a seat in a non-public room when filling in the paper and electronic questionnaire forms at the hospital. All acquired data were stored locally in a pseudonymized format and in compliance with the institution's data security standards.

### VILL Questionnaire

The VILL questionnaire is a PRO to assess the VRQoL in AMD with a particular focus on visually challenging conditions such as low luminance and low contrast. Its development and validation steps were in accordance with regulatory agencies’ requirements for PRO development to support labeling claims in drug and medical device development.^9^ The VILL consists of three subscales with 33 items in total, each providing four response categories and a “not applicable” option (Terheyden JH, Pondorfer SG, Behning C, et al. Disease-specific assessment of vision impairment in low luminance in age-related macular degeneration - a MACUSTAR study report. *Br J Ophthalmol.* 2022; doi:10.1136/bjophthalmol-2021-320848). Its three subscales include “reading and accessing information” (17 items), “mobility and safety” (12 items), and “emotional well-being” (four items). The VILL-33 questionnaire has been shown to be a reliable and valid instrument with good discriminatory properties between AMD stages (Terheyden JH, Pondorfer SG, Behning C, et al. Disease-specific assessment of vision impairment in low luminance in age-related macular degeneration - a MACUSTAR study report. *Br J Ophthalmol.* 2022; doi:10.1136/bjophthalmol-2021-320848) and significant associations with functional tests including low-luminance visual acuity, contrast sensitivity, and retinal sensitivity under mesopic and scotopic conditions (Terheyden et al., EURETINA 2021, conference abstract).

### Psychometric Evaluation and Statistical Analysis

Pseudo-interval-scaled person measures were calculated from the questionnaire responses using Rasch analysis, with high person measures indicating high vision-related quality of life. For this purpose, we anchored item parameters obtained from a randomly selected administration mode per participant to obtain the overall latent trait model. The random sample was selected to have the same distribution of all administration modes in the full dataset (45% paper administrations, 35% interview administrations, and 20% electronic administrations).^13^ We then examined if the Rasch model requirements were met with the dataset as outlined previously.^12^^,^^14^^,^^15^ When items had infit or outfit mean-square values > 1.5 or < 0.5, misfitting item responses were removed and item fit was re-investigated as a measure of quality control.^16^ Differential item functioning by mode of administration was investigated based on the random sample of all available VILL questionnaire administrations explained above.

For the analysis of equivalence among the modes of administration, we followed recommendations by the International Society for Pharmacoeconomics and Outcomes Research (ISPOR).^17^ To investigate the differences of responses across the different administration modes, we calculated intraclass correlation coefficients (ICCs) and 95% confidence intervals (CIs) of person measures and interpreted those following Cicchetti and Sparrow,^18^ with ICC values ≥ 0.75 indicating excellent agreement. We also constructed Bland–Altman plots, calculated coefficients of repeatability, and conducted Deming regression based on person measures.^19^^,^^20^ We investigated how the differences of person measures among the different modes of administration were distributed in a subgroup of participants matched by age, considering the initial mode of administration, time interval between administrations, and visual impairment or hearing difficulties. The LLQ data were also evaluated using a Rasch model to generate person measures. We scored the LLQ similar to the VILL questionnaire, with higher scores indicating higher vision-related quality of life. We calculated correlation coefficients between VILL questionnaire and LLQ person measures.

We used Winsteps software (Chicago, IL)^21^ for Rasch analysis and SPSS Statistics 25 (IBM Corporation, Chicago, IL) and R 3.6.1 (R Foundation for Statistical Computing, Vienna, Austria) for statistical analyses. Deming regression was performed using the R package *deming*. *P* < 0.05 was considered significant. We corrected for multiple testing when indicated.

## Results

After exclusion of 21 individuals from the study (≥50% missing responses, *n* = 7 [paper]; availability of only one administration mode, *n* = 7; consent withdrawal, *n* = 7), we included a total of 309 participants in the analysis (Table 1). The administration modes of the VILL questionnaire were paper for 307 participants (99%), interview for 241 participants (78%), and electronic for 135 participants (44%), adding up to a total of 683 questionnaires available for analysis. The mean number of modes of administration per participant was 2.2 ± 0.4. Among the participants, 172 (56%) reported having retinal disease, 72 (23%) reported cataracts, 70 (23%) reported glaucoma, 37 (12%) reported anterior segment disease, and 69 (22%) reported other eye conditions.

**Table 1.** Sample Characteristics

Characteristic	
Age (yr), mean ± SD	63.1 ± 13.8
Gender, *n* (%)	
Female	188 (60.8)
Male	121 (39.2)
Education, *n* (%)	
Elementary school	93 (30.1)
Secondary school	111 (35.9)
High school	33 (10.7)
University graduate	65 (21.0)
Missing data	7 (2.3)
Employment status, *n* (%)	
Working	152 (49.2)
Unemployed	20 (6.5)
Retired	126 (40.8)
Missing data	11 (3.6)
Living situation, *n* (%)	
Alone	82 (26.5)
With others	220 (71.2)
Missing data	7 (2.3)
Marital status, *n* (%)	
Married	184 (59.5)
Widowed	44 (14.2)
Divorced	45 (14.6)
Unmarried	35 (11.3)
Missing data	1 (0.3)
VILL questionnaire administration interval (days), mean ± SD	13 ± 14
Visual acuity (logMAR), better eye, mean ± SD	0.22 ± 0.21
Hearing difficulties, *n* (%)	75 (24.3)

### Psychometric Properties

All subscales of the VILL questionnaire had adequately functioning rating scales and a high internal consistency in our random sample of administration modes (Table 2). We initially observed misfit in three items belonging to the reading and accessing information subscale and in two items of the mobility and safety subscale. After removal of 22 misfitting item responses to the reading and accessing information subscale and of 10 misfitting item responses to the mobility and safety subscale, all items fit the Rasch model. None of the emotional well-being subscale items showed misfit. Four items showed differential item functioning (DIF), three when comparing electronic administration to paper or interview administration, respectively, and one item when comparing paper administration to interview administration (Table 2). To investigate the impact of the items showing DIF, we calculated ICCs between original person measures and modified person measures from Rasch models in which the DIF items were omitted. The respective ICCs were 0.999 (95% CI, 0.998–1.000) for the reading subscale, 0.999 (95% CI, 0.997–0.999) for the mobility subscale, and 0.975 (95% CI, 0.967–0.981) for the emotional subscale. We retained all four items showing DIF for this reason.

**Table 2.** Fit Parameters of the VILL-33 Questionnaire in a Random Sample of the Population^a^

Parameters	Rasch Model	Reading and Accessing Information	Mobility and Safety	Emotional Well-Being
Disordered thresholds	None	None	None	None
Misfitting items	0	None	None	None
Person reliability	>0.8	0.94	0.92	0.81
Person separation index	>2.0	4.09	3.42	2.08
Difference in person and item mean	<1.0	0.81	0.53	1.54
Differential item functioning (mode of administration)	<1.0 *P* > 0.05	Item 9 (paper vs. electronic)	Item 18 (interview vs. electronic)	Item 35 (paper vs. interview) Item 36 (interview vs. electronic)

a Administration modes following the frequency of occurrence in the full dataset (139 paper administrations, 109 interview administrations, 61 electronic administrations) are compared with Rasch model requirements (second column).

### Repeatability Across Modes of Administration

Intraclass correlations across different modes of administration were excellent following the interpretation suggested by Cicchetti and Sparrow (Table 3).^18^ ICCs of the reading and mobility subscales when comparing paper with interview administration (0.930 and 0.919, respectively) were significantly higher than the ICC of the emotional subscale (0.799) and higher for the reading subscale (0.967) than the emotional subscale (0.907) when comparing interview with electronic administration. The median person measure differences among the modes of administration were 2.2% for the reading subscale range (0.14 standard deviation [SD] of the distribution within our sample), 1.6% for the mobility subscale range (0.08 SD), and 4.7% (0.16 SD) for the emotional subscale range. In Deming regression of all participants, the person measures of all subscales were significantly lower for paper than interview administration, and person measures of the reading and the emotional subscale were significantly lower for paper than electronic administration of the VILL questionnaire (Table 3). Yet, the mean differences were low across modes of administration (Fig.). Correlation coefficients between VILL questionnaire and LLQ scores were not significantly different when considering varying modes of administration of the VILL questionnaire (Supplementary Table, Supplementary Fig.).

**Table 3.** Intermode Reliability Statistics of the VILL-33 Questionnaire Subscales

Subscale	Paper Versus Interview (*n* = 239)	Paper Versus Electronic (*n* = 133)	Interview Versus Electronic (*n* = 67)
**Reading and accessing information**			
ICC (95% CI)	0.930 (0.900–0.949)	0.951 (0.923–0.968)	0.967 (0.946–0.980)
Mean difference^a^	−0.35 (2.4)	−0.32 (2.2)	0.08 (0.5)
Coefficient of repeatability (% scale range)	2.14 (14.4)	1.81 (12.2)	1.52 (10.2)
Deming intercept (95% CI)	0.335 (0.194–0.475)^b^	0.192 (0.047–0.337)^b^	−0.092 (−0.281–0.097)
Deming slope (95% CI)	0.978 (0.898–1.058)	0.893 (0.809–0.976)^b^	0.979 (0.841–1.118)
**Mobility and safety**			
ICC (95% CI)	0.919 (0.887–0.941)	0.959 (0.941–0.971)	0.955 (0.926–0.972)
Mean difference^a^	−0.41 (3.0)	−0.18 (1.3)	0.22 (1.6)
Coefficient of repeatability (% scale range)	2.67 (19.7)	1.80 (13.3)	1.79 (13.2)
Deming intercept (95% CI)	0.422 (0.225–0.620)^b^	0.143 (−0.041–0.327)	−0.176 (−0.439–0.087)
Deming slope (95% CI)	1.018 (0.929–1.107)	0.966 (0.882–1.050)	1.058 (0.923–1.193)
**Emotional well-being**			
ICC (95% CI)	0.799 (0.642–0.875)	0.916 (0.871–0.944)	0.907 (0.847–0.944)
Mean difference^a^	−1.74 (11.9)	−0.68 (4.7)	0.63 (4.3)
Coefficient of repeatability (% scale range)	6.57 (45.1)	3.97 (27.2)	4.69 (32.2)
Deming intercept (95% CI)	1.608 (1.089–2.127)^b^	0.737 (0.299–1.174)^b^	−0.325 (−1.001–0.350)
Deming slope (95% CI)	0.937 (0.844–1.030)	1.029 (0.934–1.123)	1.192 (1.018–1.366)^b^

a Positive differences indicate higher person measures with paper administration than interviewer administration (first column), with paper administration than electronic administration (second column), or with interviewer administration than electronic administration (third column)

b These 95% CIs exclude the intercept 0 (potential systematic difference between measurements) or slope 1 (potential proportional difference between measurements).

**Figure.**
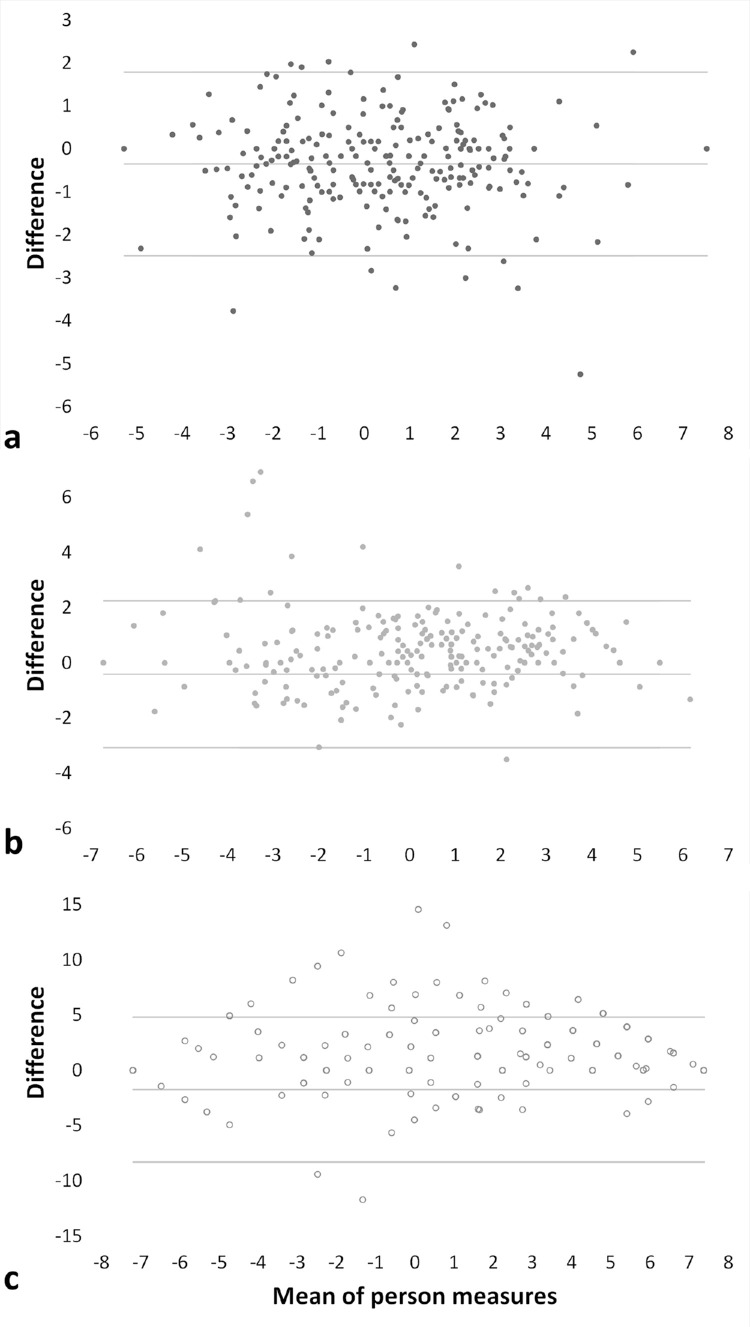
Bland–Altman plots comparing self-administration via paper questionnaires with interviewer administration with respect to the VILL questionnaire subscales of (a) reading and accessing information, (b) mobility and safety, and (c) emotional well-being. The mean differences among the different modes of administration are displayed on the vertical axis. Positive differences indicate higher person measures with paper self-administration than interviewer administration in logits.

### Impact of Initial Mode of Administration, Interval, Visual Acuity, and Hearing

The group to which the VILL questionnaire was initially self-administered (*n* = 217) was noticeably larger than the group with initial interviewer administration (*n* = 92). Both groups differed significantly by age (*P* < 0.0001), level of education (*P* = 0.034), employment status (*P* < 0.0001), interval between the administrations (*P* = 0.007), and visual acuity (*P* = 0.015). We matched 92 participants with initial self-administration and initial interviewer administration by age. This resulted in none of the above-mentioned variables showing any significant differences in the subcohort (*P* ≥ 0.139).

These 184 participants were subsequently included in a subgroup analysis, in which we additionally investigated the impact of the initial mode of administration and other potential confounding variables on the overall repeatabilities across mode of administration. Besides initial administration mode, we investigated how the variables administration interval, best-corrected visual acuity of the better eye, and self-reported hearing difficulties were associated with the person measure differences between administration modes (Table 4). The overall differences of person measures among the different modes of administration in the subgroup were consistent with the results of the overall group (Table 3). None of the mentioned factors was significantly associated with differences among the modes of administration when correcting for multiple testing, using the Bonferroni–Holm method (corrected *P* ≥ 0.11) (Table 4).

**Table 4.** Associations Between VILL-33 Questionnaire Subscale Person Measure Differences and Modes of Administration and Sample Characteristics in a Subsample (*n* = 184)^a^

	Person Measure Differences		
	Paper Versus Interview (*n* = 173)	Paper Versus Electronic (*n* = 67)	Interview Versus Electronic (n = 56)
**Reading and accessing information**			
Overall, mean ± SD	−0.43 ± 1.15	−0.27 ± 0.93	0.12 ± 0.81
Initial administration mode, mean ± SD			
Interviewer	−0.21 ± 1.10	−0.51 ± 1.01	−0.22 ± 0.75
Self	−0.67 ± 1.17	−0.14 ± 0.86	0.36 ± 0.79
*P* (corrected)	0.011 (0.385)	0.234 (1.0)	0.003 (0.108)
Administration interval, mean ± SD			
<2 wk	−0.33 ± 1.09	−0.39 ± 0.91	0.06 ± 0.92
≥2 wk	−0.38 ± 1.10	−0.25 ± 0.94	0.09 ± 0.72
*P* (corrected)	0.824 (1.0)	0.235 (1.0)	0.701 (1.0)
Visual acuity (logMAR), better eye^b^			
Pearson's *r*	0.003	0.180	−0.050
*P* (corrected)	0.966 (1.0)	0.144 (1.0)	0.712 (1.0)
Hearing difficulties, mean ± SD			
Reported	−0.29 ± 1.27	−0.39 ± 0.81	−0.08 ± 0.93
Not reported	−0.47 ± 1.12	−0.27 ± 0.96	0.17 ± 0.79
*P* (corrected)	0.437 (1.0)	0.445 (1.0)	0.837 (1.0)
**Mobility and safety**			
Overall, mean ± SD	−0.49 ± 1.46	−0.12 ± 0.92	0.22 ± 0.97
Initial administration mode, mean ± SD			
Interviewer	−0.40 ± 1.68	0.02 ± 0.89	0.32 ± 0.90
Self	−0.59 ± 1.16	−0.19 ± 0.94	0.14 ± 1.03
*P* (corrected)	0.104 (1.0)	0.328 (1.0)	0.281 (1.0)
Administration interval, mean ± SD			
<2 wk	−0.39 ± 1.41	−0.20 ± 0.90	−0.12 ± 0.73
≥2 wk	−0.45 ± 1.29	−0.16 ± 0.94	0.36 ± 0.95
*P* (corrected)	0.636 (1.0)	0.472 (1.0)	0.025 (0.850)
Visual acuity (logMAR), better eye^b^			
Pearson's *r*	−0.095	0.049	−0.186
*P* (corrected)	0.214 (1.0)	0.691 (1.0)	0.170 (1.0)
Hearing difficulties, mean ± SD			
Reported	−0.70 ± 1.60	−0.23 ± 1.10	0.02 ± 0.68
Not reported	−0.42 ± 1.42	−0.13 ± 0.85	0.26 ± 1.03
*P* (corrected)	0.198 (1.0)	0.383 (1.0)	0.273 (1.0)
**Emotional well-being**			
Overall, mean ± SD	−1.92 ± 3.55	−1.01 ± 2.43	0.60 ± 2.50
Initial administration mode, mean ± SD			
Interviewer	−1.57 ± 4.05	−0.52 ± 1.52	0.66 ± 2.87
Self	−2.30 ± 2.86	−1.27 ± 2.76	0.55 ± 2.25
*P* (corrected)	0.136 (1.0)	0.069 (1.0)	0.967 (1.0)
Administration interval, mean ± SD			
<2 wk	−1.59 ± 3.18	−0.66 ± 1.70	−0.10 ± 2.05
≥2 wk	−1.99 ± 3.62	−0.71 ± 2.34	0.94 ± 2.48
*P* (corrected)	0.413 (1.0)	0.369 (1.0)	0.154 (1.0)
Visual acuity (logMAR), better eye^b^			
Pearson's *r*	−0.102	−0.109	0.019
*P* (corrected)	0.184 (1.0)	0.378 (1.0)	0.887 (1.0)
Hearing difficulties, mean ± SD			
Reported	−1.60 ± 2.96	−1.05 ± 2.23	−0.45 ± 2.30
Not reported	−2.01 ± 3.74	−1.11 ± 2.43	0.85 ± 2.50
*P* (corrected)	0.493 (1.0)	0.987 (1.0)	0.232 (1.0)

a Initial interviewer administration or initial self-administration is matched by age. Positive differences indicate higher person measures with paper administration than interviewer administration (first column), with paper administration than electronic administration (second column), or with interviewer administration than electronic administration (third column).

b Stratifying the analysis by the presence of any visual impairment (best-corrected visual acuity logMAR ≥ 0.2) detected no effect modification (*P* ≥ 0.315, Bonferroni–Holm corrected).

## Discussion

In this study, we found VILL questionnaire responses to be overall unaffected by the mode of administration and that interviewer administration, paper-and-pencil self-administration, and electronic self-administration of the VILL questionnaire can be considered equivalent. The initial mode of administration, administration interval up to 10 weeks, visual acuity of the better seeing eye, or hearing difficulties did not impact results, indicating that the VILL questionnaire is a robust instrument across different modes of administration and a diverse set of respondents. The high correlation of the VILL questionnaire to the LLQ further supports its criterion validity across all administration modes.

The intermode reliability of emotional subscale scores was slightly less compared to the reading and mobility subscale scores, and there was a tendency toward reporting lower vision-related quality of life in paper administrations compared to interviews and electronic administrations of the VILL questionnaire. This is in agreement with the lower reported quality of life for paper compared with interview administrations in the literature, which may be explained by respondents providing answers that are more socially accepted in interview contexts.^22^^–^^24^ However, the differences among modes of administrations in our study were below a level commonly considered clinically meaningful. Coons et al.^17^ suggested an ICC value ≥ 0.7 as the cut-off for score levels being interpreted at a group level, which was met with all modes of administration of the VILL questionnaire in our study.

The test–retest reliability of the VILL questionnaire has previously been investigated in the context of the MACUSTAR study. Mean differences between test and retest administrations of the VILL questionnaire were 2.1% of the reading subscale range, 0.5% of the mobility subscale range, and 4.5% of the emotional subscale range (Terheyden JH, Pondorfer SG, Behning C, et al. Disease-specific assessment of vision impairment in low luminance in age-related macular degeneration - a MACUSTAR study report. *Br J Ophthalmol.* 2022; doi:10.1136/bjophthalmol-2021-320848). The respective median differences among administration modes in this study were in a similar range (2.2%, 1.6%, and 4.7% for the reading, mobility, and emotional subscales, respectively), indicating that different modes of administration of the VILL questionnaire are highly comparable. Similar to the previous results from a test–retest setting, the emotional subscale was noticeably less repeatable than the other subscales in this study. For content validity, we retained the emotional subscale to be further evaluated. The ISPOR has previously suggested interpreting effect sizes between 0.2 and 0.49 SD as meaningful in absence of an established minimally important difference.^17^ The median differences of the VILL are below this range of clinically meaningful change, which further supports the suggestion that observed statistical differences are not clinically relevant (0.14, 0.08, and 0.16 SD for the reading, mobility, and emotional subscales, respectively). Previous research also suggests that most PRO measures reach a score difference of <5% of the scale range when comparing self-administrated questionnaires using electronic versus paper-and-pencil forms.^25^ The respective differences in the reading, mobility, and emotional subscales of the VILL questionnaire were 2.2%, 1.3%, and 4.7%, supporting the suggestion that electronic VILL can be considered equivalent to its paper-and-pencil version.

Clayton and colleagues^8^ have published work on differences among the administration modes in specific ophthalmic PROs, focusing on ocular surface disease. They investigated differences between electronic and paper-and-pencil administration of the Refractive Error Quality of Life Instrument, Ocular Surface Disease Index, and Visual Function Questionnaire (driving questions) and did not identify any differences they considered clinically significant (≤2.1% of scale range). Rutherford et al.^26^ found self-assessment via paper-and-pencil forms and digitally to be equivalent in a meta-analysis that included the results of 56 papers across specialities. Even though self-administration was also equivalent to interviewer administration in their study, they argued that an interview setting may introduce a certain amount of bias compared to any form of self-administration. Other studies have investigated how general health-related quality of life instruments are affected by the selection of their mode of administration, and the reported effects were mostly negligible.^27^^,^^28^ Similar to these reports, we have found paper-based or electronic self-assessment to be comparable with interviewer administration of the VILL questionnaire.

The strengths of our study include its relatively large number of participants, the highly standardized assessment including administration conditions and the use of trained interviewers, and the comprehensive set of analyses performed. We have also included an analysis comparing the VILL questionnaire with the LLQ for the first time, to the best of our knowledge. As outlined previously, the scoring of the VILL is based on latent trait models, which makes it less prone to error due to outliers or missing data. The VILL questionnaire itself has been developed on the basis of qualitative and quantitative research work, as recommended by regulatory agencies.^9^^,^^29^ Our analysis followed recommendations by the ISPOR,^17^ meeting available reference standards for the assessment of comparability of modes of administration. Nevertheless, our study has a number of limitations, including the lack of randomization for the initial mode of administration. We performed a subgroup analysis in order to account for this possible bias introduced by the design, but none of the participants chose the electronic PRO as the initial mode of administration, which precluded us from performing full sensitivity analyses. However, subgroup analyses suggested no impact of first mode of administration or other potential confounders such as recall bias. Additionally, heterogeneity may have been introduced by allowing participants to reply to interviews in person or via phone, complete paper questionnaires under hospital conditions or at their homes, and to complete the electronic PRO using a desktop or tablet computer. Despite the fact that the VILL questionnaire has been developed as an instrument for individuals with AMD, we have included individuals with a variety of ophthalmic conditions. The content of the VILL questionnaire items focuses on low-luminance/low-contrast situations rather than AMD specifically. The collected data were supportive of the reliability and validity of the VILL questionnaire in this cohort, and the presence or absence of AMD was not significantly associated with any of the differences among VILL questionnaire scores when comparing different modes of administration (*P* ≥ 0.075). Individuals without visual impairment were included, but an analysis stratified by the presence of visual impairment did not indicate any effect modification.

In summary, the VILL questionnaire yields comparable results across different modes of administration that are not impacted by better eye visual acuity or hearing difficulties. This further indicates that the VILL questionnaire could serve as a robust PRO measure for clinical trials in AMD.

## Supplementary Material

Supplement 1
