# Supplementary material for: Interviewer Administration Corresponds to Self-Administration of the Vision Impairment in Low Luminance (VILL) Questionnaire
Source: Transl Vis Sci Technol. 2022 Apr 21;11(4):21. doi: 10.1167/tvst.11.4.21 (PMC9034722; doi:10.1167/tvst.11.4.21)

Interviewer administration corresponds to self-administration of the Vision Impairment in Low Luminance (VILL) questionnaire

Jan Henrik Terheyden, MD; Liza Mekschrat; Reglind Ost; Gamze Bildik; Moritz Berger, PhD;

Maximilian W.M. Wintergerst, MD; Frank G. Holz, MD; Robert P. Finger, MD, PhD

– SUPPLEMENT –

Supplementary Table: Pearson correlation coefficients [95% confidence intervals] between different modes of administration of the VILL and the LLQ

VILL subscales	Correlation with LLQ		
	Paper administration	Interview administration	Electronic administration
Reading and accessing information	0.833 [0.791;0.867]	0.818 [0.770;0.857]	0.813 [0.733;0.872]
Mobility and safety	0.856 [0.820;0.886]	0.842 [0.799;0.876]	0.844 [0.776;0.894]
Emotional well-being	0.628 [0.547;0.697]	0.462 [0.353;0.558]	0.565 [0.411;0.687]

LLQ, Low Luminance Questionnaire; VILL, Vision Impairment in Low Luminance Questionnaire

Supplementary Figure: Associations between the VILL subscales Reading and accessing information, Mobility and safety, Emotional well-being and the LLQ. The VILL scores used in this plot were obtained based on a random selection procedure explained in the manuscript

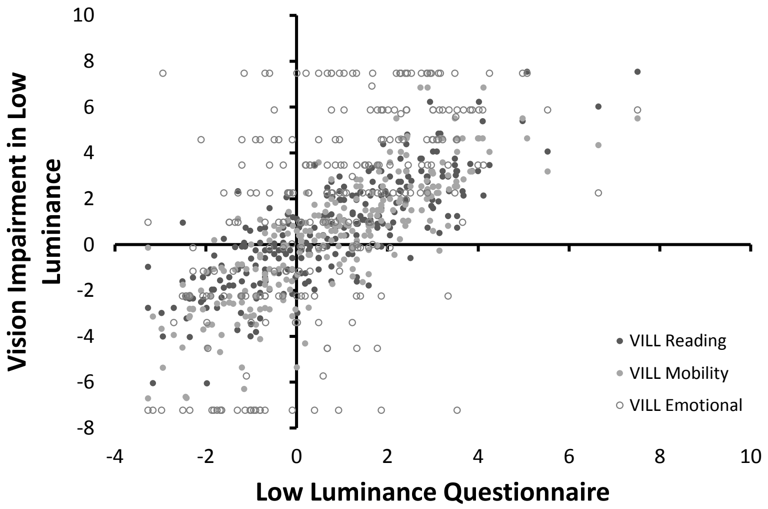

Supplement: Supplement 1 [file tvst-11-4-21_s001.pdf]
